# Optimization Design of Drilling Fluid Chemical Formula Based on Artificial Intelligence

**DOI:** 10.1155/2022/5465816

**Published:** 2022-10-04

**Authors:** Li Chen

**Affiliations:** College of Chemical Engineering, Yangzhou Polytechnic Institute, Yangzhou 225127, China

## Abstract

Through the research and development of the regression prediction function of support vector machine, this paper applies it to the prediction of drilling fluid performance parameters and the formulation design of drilling fluid. The research in this paper can reduce the experimental workload and improve the efficiency of drilling fluid formulation design. The apparent viscosity (*AV*), plastic viscosity (*PV*), API filter loss (*FL*_API_), and roll recovery (*R*) of the drilling fluid were selected as the inspection objects of the drilling fluid performance parameters, and the support vector machine was used to establish a model for predicting the drilling fluid performance parameters. This predictive model was used as part of the overall drilling fluid formulation optimization design model. For a given drilling fluid performance parameter requirement, this model can be applied to reverse the addition of various treatment agents, and finally, the prediction accuracy of the model is verified by experiments.

## 1. Introduction

As a main tool, computer is introduced into the design and management of drilling fluid engineering. By combining computer technology with the thinking of drilling fluid experts, the level of drilling fluid design can be raised to a new level, and the design speed and quality will be greatly improved [1–5]. The development of the drilling fluid optimization design system can not only solve the problems in the traditional drilling fluid design but also has a more prominent feature that the computer system can store the design data for secondary use so that the experience accumulated in the previous design can be absorbed and the mistakes made in the past can be avoided in the new drilling fluid design process [6–9]. At the same time, the system can also output a unified design document. The research on the drilling fluid optimization design system, the establishment of a high-level drilling fluid database, and the development of efficient drilling fluid optimization design methods will contribute to the learning and promotion of successful drilling fluid design experience, the realization of integrated management of formulas, the improvement of information utilization, the integration of modern computer technology and drilling fluid design, and the realization of automation, standardization, and intelligence of drilling fluid design.

The research of drilling fluid optimization design system can collect and popularize the successful drilling fluid design case experience summarized by the previous drilling, guide the new technicians to conduct drilling fluid design, and continuously promote the improvement of drilling fluid design technology.

Based on the research of case-based reasoning technology, rule-based reasoning technology, and support vector machine regression prediction technology, this paper also realizes their fusion reasoning. It not only avoids the disadvantages of each reasoning model operating in isolation and cannot fully apply the relevant conclusions in each other's reasoning to improve the reasoning success rate but also realizes the complementary advantages of each reasoning model and improves the design success rate of the system.

## 2. The Concept of Support Vector Machine

The support vector machine (SVM) is based on the Vapnik-Chervonenkis dimension of statistical theory and the structural risk minimization principle. It seeks the best compromise between model complexity (learning accuracy for a specific training sample) and learning ability (ability to identify random samples without error) based on limited sample information to expect the best generalization ability. The most significant difference between it and the neural network is that it only needs to build a support vector machine model based on limited training samples by mining the corresponding relationship between the input and output data, to realize the prediction of unknown data. Support vector machines not only perform well in processing language, text, face recognition, etc., but also achieve good results in regression, such as using logging data to predict formation porosity and reservoir properties in the field of well logging [10–13]. Support vector machine is influencing various areas of machine learning through this new method of intelligent machine learning. Support vector machines originated from solving classification problems. The support vector machine introduces an insensitive loss function to solve the regression estimation problem of linear and nonlinear systems, which also achieves the same effect as the classification problem. Based on the principle of the support vector machine, this section will gradually explain the regression prediction principle of the support vector machine in detail.

### 2.1. Basic Theory

The basic idea of statistical learning theory is to estimate limited or small-scale sample data, mainly to study the relationship between experience minimization and empirical risk, expected risk, and how to seek new learning methods and principles based on existing ones. Statistical learning theory has apparent advantages in studying the learning laws of limited samples. It also effectively avoids the shortcomings of traditional statistical theory that quickly make the model fall into the local minimum due to overfitting and too many dimensions. Its progressive nature makes statistical theory develop rapidly under the efforts of many researchers [14, 15].

An essential concept in statistical theory, the Vapnik-Chervonenkis dimension (VC dimension), can measure the generalization ability of the model trained by the support vector machine [16–19]. Under limited training samples, the larger the VC dimension of the learning machine, the more complex the learning machine will be, and the larger the confidence interval will be, which will eventually lead to a larger gap between the actual risk and the empirical risk, which means the model is more generalizable.

If there is a sample set with *n* data samples, which can be separated by a function set in all possible 2^*n*^ ways, then the function set is said to be able to break up the sample set with *n* samples. Therefore, the VC dimension of the indicator function set is the maximum number of sample sets that can be broken up. In short, if there are *n* samples of sample sets that this function set can separate, and this function set cannot separate n + 1 samples of sample sets, the dimension of the function set is *n*. In particular, if a corresponding function set can be found to separate the sample set of any number of samples, then the VC dimension of this function set is infinite. The VC dimension of the general function set can be defined based on the indicator function VC dimension. The basic principle is to define a threshold to convert a real-valued function into a binary indicator function.

Besides VC dimension theory, structural risk minimization is the second factor that has a great impact on machine learning. To achieve better generalization ability in machine learning, the traditional theory reduces the empirical risk to make it reach the minimum value. Based on statistical learning theory, it is found that the quality of generalization ability is also related to the VC dimension, which is used to narrow the confidence range. Since there have been many shortcomings in the past in relying on empirical risk to evaluate the generalization ability of learning machines, Vapnik et al. proposed the method of applying structural risk minimization to solve this problem when they studied support vector machines. The basic idea of structural risk minimization theory is to arrange the function set into a sequence of subsets in order of VC dimension size and then minimize the actual risk by calculating each subset's empirical risk and confidence range.

One of the ideas to achieve structural risk minimization is to design a particular structure of the function set so that each subset can achieve the minor empirical risk (such as making the training error 0) and then select the appropriate subset to minimize the confidence range. The function that minimizes the empirical risk in this subset is the optimal function. The support vector machine method is a concrete realization of this idea.

### 2.2. Classification

In the period of popular application of neural network systems, some scholars began to study the machine learning method with limited samples and first proposed the theory of statistical knowledge [20]. With the continuous progress in machine learning, new approaches are emerging. At the same time, it has been found that neural networks also have some drawbacks in dealing with practical problems, such as overlearning, underfitting, the curse of dimensionality, and falling into a local minimum. It is also not suitable for small samples of drilling fluid experimental data. With the continuous efforts of researchers, support vector machine theory has been paid more and more attention and developed rapidly with its unique processing methods for limited sample problems, nonlinear problems, and high-dimensional recognition problems.

In the early days of the emergence of support vector machines, it was considered that only two classification problems could be handled. Its basic idea was to find an optimal classification hyperplane to divide the data samples. Later, as classification requirements increased, support vector machines were developed to handle multiclassification problems [21–23]. The classification problem theory will be introduced in the following.

Suppose there are linearly separable samples, as shown in the formula given below:

(1) $$\begin{array}{c} \left(x_{i},y_{i}\right),x_{i}\in R^{\left(N\right)},y_{i}\in \left\{+a,-a\right\},i=1,2,\ldots l\text{.} \end{array}$$

Since the sample is linearly separable, it can be expressed as *y* = +*a* or *y* = −*a*. If *x*_*i*_ belongs to the first category, *y* = +*a*; otherwise, *y* = −*a*. The basic idea of the support vector machine classification machine is to introduce a classification plane to separate the two samples as accurately as possible. If the classification plane found can completely separate the two types of samples and produce the most significant classification distance, then this plane is called the optimal separating hyperplane.

The optimal separating hyperplane is expressed as follows:

(2) $$\begin{array}{c} \omega \cdot x+b=0\text{.} \end{array}$$

Since these two types of samples are linearly separable, they satisfy the relationship of formulas (3)-(4):

(3) $$\begin{array}{c} \omega \cdot x_{i}+b\geq a,y_{i}=\pm a,i=1,2,3\ldots l\text{.} \end{array}$$

In the formula, *ω* · *x*_*i*_ are the inner product of two vectors. If the values of *ω* and *b* are appropriately adjusted, then the support vector that satisfies the formula (3) and is the closest point to the hyperplane (the point that falls on the two dashed lines) can be calculated.

According to the definition of the optimal separating hyperplane, its decision function is obtained as shown in the following formula:

(4) $$\begin{array}{c} f\left(x\right)=sign\left(w\cdot x+b\right)\text{.} \end{array}$$

Convert the optimal hyperplane into a quadratic programming problem solution, as shown in formula given below:

(5) $$\begin{array}{c} \begin{array}{c} \mathrm{Min}\;\frac{1}{2}||\omega {||}^{2}\\ s.t.\;y_{i}\left(w\cdot x+b\right)\geq 0,i=1,2,\ldots l \end{array}\text{.} \end{array}$$

The method described above is where the data samples are linearly separable. Still, if the vector distribution is linearly inseparable, then slack variables must be introduced to solve this problem [24–26]. The specific method is to take a positive number for the introduced slack variable, select a nonlinear mapping function *ϕ*(*x*), and convert the original problem from a two-dimensional to a high-dimensional space for processing so that the nonlinear samples can be linearly divided in the high-dimensional space.

To avoid the cumbersome inner product calculation in high-dimensional space, the concept of kernel function can be introduced to replace the internal product operation so that the calculation amount is no longer proportional to the space dimension, which significantly improves the calculation efficiency. This paper uses the radial basis function as the kernel function, so the nonlinear optimization classification method becomes

(6) $$\begin{array}{c} \begin{array}{c} \frac{1}{2}||\omega {||}^{2}+C\overset{l}{\underset{i=1}{\sum }}\;\xi_{i}\\ y_{i}\left(\omega \cdot \varphi \left(x_{i}\right)+b\right)\geq 1-\xi_{i}\\ \xi_{i}\geq 0,i=1,2,\ldots l \end{array}\text{.} \end{array}$$

Its corresponding dual form is as follows:

(7) $$\begin{array}{c} \begin{array}{c} \mathrm{Max}\;\overset{l}{\underset{i=1}{\sum }}\;a_{i}-\frac{1}{2}\overset{l}{\underset{i=1}{\sum }}\;\overset{l}{\underset{j=1}{\sum }}\;a_{i}a_{j}y_{i}y_{j}K\left(x_{i}\cdot x_{j}\right)\\ \overset{l}{\underset{i=1}{\sum }}\;a_{i}y_{i}=0\\ s.t.\;0\leq a_{i}\leq C,i=1,2\ldots l \end{array}\text{.} \end{array}$$

From the KKT (Karush–Kuhn–Tucker) condition, we can get the formula given below:

(8) $$\begin{array}{c} \begin{array}{c} a_{i}\left[y_{i}\left(\omega \cdot \varphi \left(x_{i}\right)+b-1+\xi_{i}\right)\right]=0\\ \left(C-\alpha_{i}\right)\xi_{i}=0,i=1,2,l \end{array}\text{.} \end{array}$$

From formulas (7) and (8), algebraic formula for *b* can be obtained, which is given below:

(9) $$\begin{array}{c} \begin{array}{c} y_{i}\left(\sum a_{j}y_{j}K\left(x_{i},x_{j}\right)+b-1=0\right)\\ s.t.\;0\leq a_{i}\leq C \end{array}\text{.} \end{array}$$

By bringing formula (8) into the support vector, *b* can be obtained, and finally, the classification function is obtained, which is given below:

(10) $$\begin{array}{c} f\left(x\right)=sign\left(\overset{l}{\underset{i=1}{\sum }}\;a_{i}y_{i}K\left(x_{i},x\right)+b\right)\text{.} \end{array}$$

### 2.3. Regression Prediction

With the continuous expansion of the application scope of support vector machines in classification problems, people began to explore their application methods for regression prediction of problems [27, 28]. In this section, the regression principle of the support vector machine will be described in detail.

In the support vector machine processing regression prediction problem, the value of the output result may cover the entire real number domain and is no longer as single as the classification problem. The most intuitive description of the regression prediction problem is that the support vector machine establishes the correspondence between the input data *X* and the output result *Y* through the given training samples and then uses this correspondence to predict the unknown data. At the same time, the model can be trained repeatedly so that the support vector machine has the self-learning ability.

During the training and learning process, the SVM finds a specific function, which enables it to find the correspondence between any input and the corresponding output data. The loss function is defined in the support vector machine regression machine. In statistics, the loss function is a function to measure the loss and the degree of error. The more common applications are the Huber loss function, the quadratic loss function, and the insensitive loss function. Compared with other loss functions, the insensitive loss function has fewer support vectors, reducing the calculation amount, and is the most widely used.

Suppose there is a set of data sample sets {(*x*_1_, *y*_1_), (*x*_2_, *y*_2_), (*x*_3_, *y*_3_),…, (*x*_*n*_2__*y*_*n*_)}, *x*_*i*_ ∈ *R*^*n*^, *y*_*i*_ ∈ *R*^*n*^, then the insensitive loss function selected in this paper is expressed as formula given below:

(11) $$\begin{array}{c} L\left(x_{i},y_{i},\varepsilon \right)={\left|y_{i}-f\left(x_{i}\right)\right|}_{\varepsilon }=\mathrm{Max}\left\{0,\left|y_{i}-f\left(x_{i}\right)\right|-\varepsilon \right\}\text{.} \end{array}$$

Given a set of training samples (*x*_*k*_, *y*_*k*_), *k*=1,2,3 … *n*, the regression problem is establishing a function correspondence between *x* and *y* through the given training samples, *y* *=* *f (x)*, which satisfies the minimum insensitive loss function. When the difference of *y* *=* *f (x*_*i*_) between *y*_*i*_ is less than the defined insensitive loss function *ε*, the error is not included in the loss function. The principles of linear and nonlinear regression will be introduced separately below.

#### 2.3.1. Linear Regression Model of Support Vector Machine

In linear regression [29–31], the insensitive loss function of a certain precision is defined to satisfy *ε* ≥ 0 and relaxation factors *ξ*_*k*_ ≥ 0*ξ*_*k*_^*∗*^ ≥ 0 and parameter *C* are introduced (penalty factor *C* meets *C* ≥ 0, indicating the degree of penalty for samples exceeding *ε*). The problem of the optimal hyperplane that is difficult to solve is transformed into an easy-to-implement quadratic programming problem. The objective function is as follows:

(12) $$\begin{array}{c} \begin{array}{c} \mathrm{Min}\;\phi \left(w,b\right)=\frac{1}{2}||w{||}^{2}+C\overset{n}{\underset{k=1}{\sum }}\;\left(\xi_{k}+\xi_{k}^{*}\right)\\ s.t.\;y_{k}-w\cdot x_{k}+b\leq \varepsilon +\xi_{k}\\ w\cdot x_{k}-b-y_{k}\leq \varepsilon +\xi_{k}^{*},k=1,2,\ldots ,n \end{array}\text{.} \end{array}$$

The first term in the formula makes the function smoother and improves the model's generalization ability, and the second term reduces the model error. The introduction of the penalty factor *c* balances these two terms. After introducing the Lagrange multipliers, *α*,*α*^*∗*^ and Lagrange functions, (12) becomes:

(13) $$\begin{array}{c} \begin{array}{c} L\left(w,b,\alpha ,\alpha^{*},\xi_{k},\xi_{k}^{*},\eta_{k},\eta_{k}^{*}\right)=\frac{1}{2}||w{||}^{2}+C\overset{n}{\underset{k=1}{\sum }}\;\left(\xi_{k}+\xi_{k}^{*}\right)\\ -\overset{n}{\underset{k=1}{\sum }}\;\alpha_{k}\left(\varepsilon +\xi_{k}-y_{k}+w\cdot x_{k}-b\right)\\ -\overset{n}{\underset{k=1}{\sum }}\;\alpha_{k}^{*}\left(\varepsilon +\xi_{k}^{*}+y_{k}-w\cdot x_{k}+b\right)\\ -\overset{n}{\underset{k=1}{\sum }}\;\left(\eta_{k}\xi_{k}+\eta_{k}^{*}\xi_{k}^{*}\right) \end{array}\text{.} \end{array}$$

Solving the above Lagrange problem, the dual problem is obtained as follows:

(14) $$\begin{array}{c} \begin{array}{c} \mathrm{Max}Q\left(\alpha ,\alpha^{*}\right)=-\frac{1}{2}\overset{n}{\underset{k,s=1}{\sum }}\;\left(\alpha_{k}-\alpha_{k}^{*}\right)\left(\alpha_{s}-\alpha_{s}^{*}\right)\left(x_{k}\cdot x_{s}\right)\\ -\varepsilon \overset{n}{\underset{k=1}{\sum }}\;\left(\alpha_{k}+\alpha_{k}^{*}\right)+\overset{n}{\underset{k=1}{\sum }}\;\left(\alpha_{k}-\alpha_{k}^{*}\right)y_{k}\\ s.t.\;\left\{\begin{array}{c} \overset{n}{\underset{k,s=1}{\sum }}\;\left(\alpha_{k}-\alpha_{k}^{*}\right)=0\\ 0\leq \alpha_{k},\alpha_{k}^{*}\leq C \end{array}\right. \end{array}\text{.} \end{array}$$

Solving the above dual problem, the optimal regression decision function can be obtained as follows:

(15) $$\begin{array}{c} f\left(x\right)=\overset{n}{\underset{k=1}{\sum }}\;\left(\alpha_{i}^{*}-\alpha_{i}\right)\left(x_{i}\cdot x\right)+b\text{.} \end{array}$$

#### 2.3.2. Nonlinear Regression Model of Support Vector Machine

The method of solving the nonlinear regression problem of the support vector machine is similar to the method of dealing with the nonlinear classification problem. By mapping the original nonlinear fitting data to a high-dimensional space for calculation, for the training sample (*x*_*k*_,*y*_*k*_), *k*=1,2,…, *n*, the nonlinear regression problem is transformed into the following model:

(16) $$\begin{array}{c} \begin{array}{c} \mathrm{Min}\;\phi \left(w,\xi ,\xi^{*}\right)=\frac{1}{2}w^{T}w+C\overset{n}{\underset{i=1}{\sum }}\;\left(\xi_{k}+\xi_{k}^{*}\right)\\ st.\;y_{k}-w^{T}\varphi \left(x_{k}\right)\leq \varepsilon +\xi_{k}\\ w^{T}\varphi \left(x_{k}\right)-y_{k}\leq \varepsilon +\xi_{k}^{*}\\ \xi_{k}\geq 0,\xi_{k}^{*}\geq 0,k=1,2,\ldots ,n \end{array}\text{.} \end{array}$$

This constrained optimization problem is solved using the Lagrange multiplier method, and a kernel function is introduced, which is defined as follows:

(17) $$\begin{array}{c} K\left(x,z\right)=<\varphi \left(x\right)\cdot \varphi \left(z\right)>\text{.} \end{array}$$

Introducing this function to the solution of the dual problem, the SVM regression estimation function can be written as follows:

(18) $$\begin{array}{c} f\left(x\right)=\overset{n}{\underset{k=1}{\sum }}\;\left(\alpha_{i}^{*}-\alpha_{i}\right)K\left(x_{i},x\right)+b\text{.} \end{array}$$

## 3. Support Vector Machine Kernel Function Selection and Parameter Optimization

### 3.1. Kernel Function Selection

Support vector machine is a machine learning method based on limited samples, and its generalization ability is highly related to the selected kernel function, kernel parameter, and penalty factor *C*. The kernel function realizes the nonlinear mapping of the sample data from the input space to the feature high-dimensional space. However, it is still impossible to establish a direct relationship between the parameters and the generalization ability of the learning machine. Therefore, choosing the kernel function and parameters is a complex problem in the application field of support vector machines.

If a function can satisfy the Mercer condition, it can be used as a kernel function [25, 29]. Currently, many scholars are devoted to the research of kernel function construction. Still, so far, there is no general method to determine the kernel function, so linear kernel (LK), polynomial kernel (PK), radical basis function (RBF), and sigmoid kernel (SK) are still generally selected in practical applications. As the representative of the global kernel function, the polynomial kernel is characterized by allowing the sample points far away from the fitting function curve to influence the kernel function's value significantly. The representative of the local kernel function is the radial basis function, characterized in that the samples with farther distances have less influence on the value of the kernel function.

**Figure 1:**
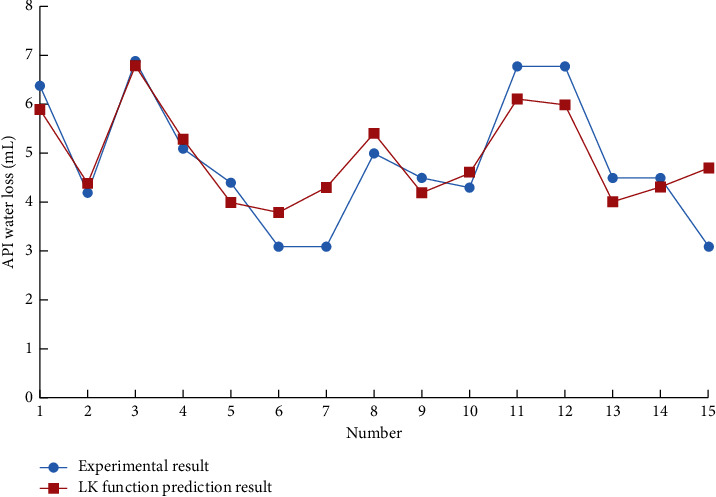
LK function prediction result.

**Figure 2:**
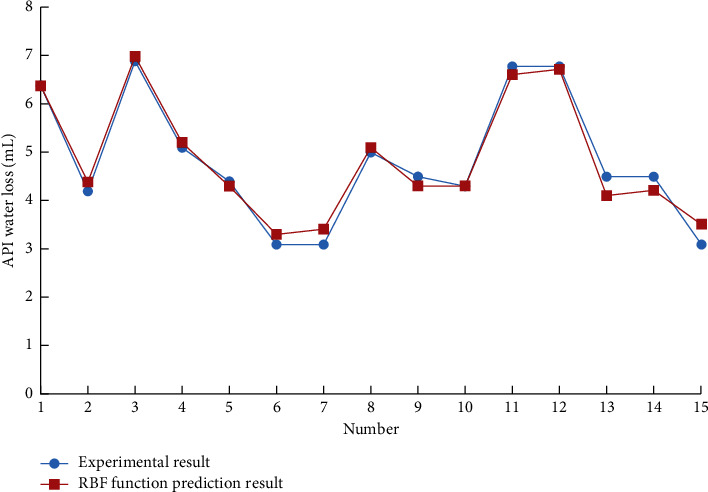
RBF function prediction result.

**Figure 3:**
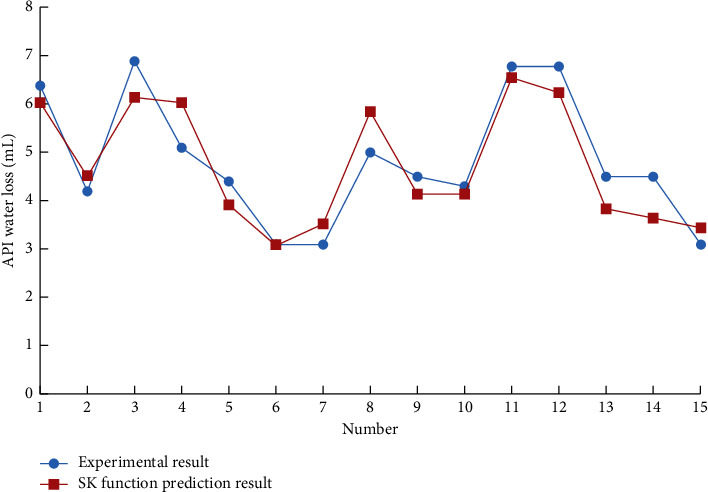
SK function prediction result.

Using the support vector machine of the drilling fluid optimization design system to predict the performance parameters of the drilling fluid, different kernel functions are used to predict the 15 groups of drilling fluid API fluid loss with other formulations. The results are shown in Figures 1–3.

The support vector machine uses the Squared correlation coefficient to measure the model's prediction accuracy. The radial basis kernel function has achieved a high data prediction accuracy, as shown in Table 1. It is found that if there is no prior understanding of the regularity of the sample data, it is more reasonable to choose the radial basis function as the kernel function of the support vector machine.

### 3.2. Kernel Parameter Optimization Method

Although the choice of the kernel function will lead to different prediction performances of the support vector machine, it is found that the selection of the kernel parameter has a more noticeable impact on the results in the practical application of the regression prediction of the support vector machine. In many cases, it plays a crucial role in the performance of the learning machine [28, 30]. Many scholars have used random search algorithms to determine nuclear parameters. The generally recognized algorithms include the particle cluster algorithm, genetic algorithm, and ant colony optimization. Although these random search algorithms that have been developed can accurately calculate the optimal kernel parameters of support vector machines, there are some problems in application. For example, the parameter optimization process of the genetic algorithm needs to go through generations of evolutionary calculus to determine the optimal parameters, so these methods still require a high amount of training for the support vector machine.

The grid search is one of the most direct kernel parameter optimization methods. Its fundamental theory is to divide the parameters to be searched into several grids within a specific range and find the optimal parameters by traversing all the points in the grid. This method can find the optimal global solution when the optimization interval is large enough, and the step size is small enough. At the same time, the grid search method is easy to implement and easy to use. Therefore, this paper selects the radial basis function as the kernel function of the support vector machine and uses the grid search to determine the kernel parameters. The specific process is given below.

**Figure 4:**
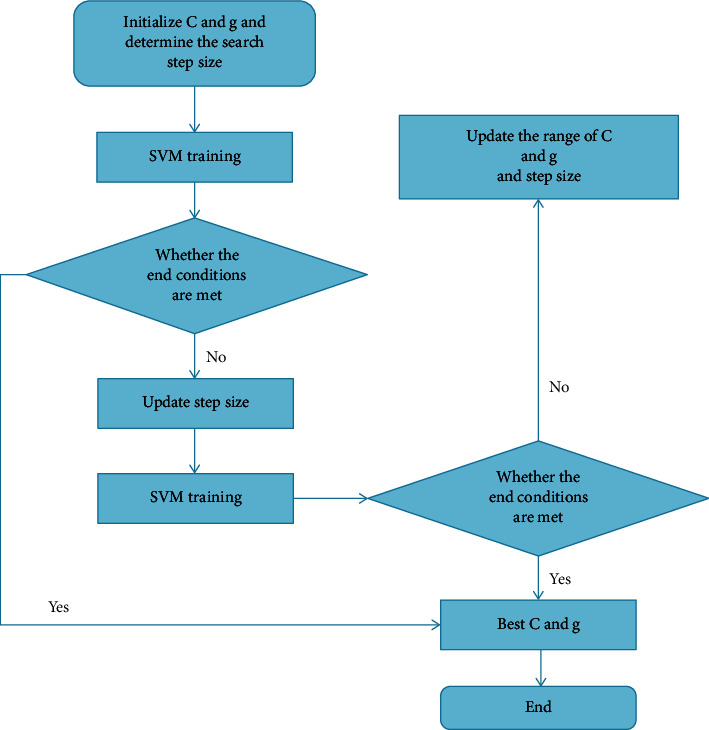
Parameter optimization of grid search.

For the penalty factor *C* and kernel function parameter *g* that need to be determined, all possible values of *C* and *g* are used as the range of grid search, and the grid of values of *C* and *g* is discretized. Then, with fixed step size, the grid is generated along the different growth directions of the two parameters *C* and *g*, which are represented by nodes in the grid. First, choose a rough search in an extensive range, and then finely search around the optimal value. Using the cross-validation method, the training data is divided into *n* subsets of the same size, and the *n* − 1 subsets are used as training samples to obtain a decision function, which is used to predict the subset that has not participated in the training. This cycle is repeated *n* times until all subsets are predicted as test samples. Take the average accuracy obtained from *n* predictions as the final accuracy, as shown in Figure 4. Studies have shown that exponentially growing grids are a reasonable and efficient search method.

## 4. Case Study

According to the above analysis of the support vector machine, since the influence of the drilling fluid treatment agent on the performance of drilling fluid is multifaceted, the performance data of the three treatment agents added to the drilling fluid were measured in the laboratory. Using the data based on support vector machine, a calculation model of a multifactor nonlinear problem is established based on the requirements of drilling fluid performance. Using this model, the drilling fluid formula that meets the requirements can be quickly calculated.

In this paper, the radial basis function is selected as the kernel function, vb.net is used to design the program, and the grid search algorithm is used to realize the optimization of model parameters, to establish a model for predicting the dosage of drilling fluid treatment agent based on support vector machine.

**Figure 5:**
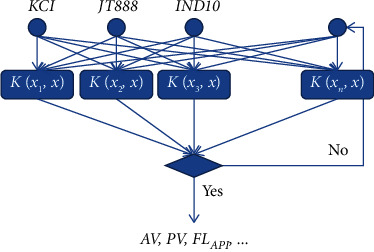
SVM prediction model structure.

Taking the commonly used strong inhibitory water-based drilling fluid in an oilfield as an example, the formula is 4% bentonite + 0.2% Na_2_CO_3_ + 1%KOH + 2%SMP-2 + 2%SPNH + coating agent + fluid loss agent + 0.3%CaO + inhibitor + 0.5%CMC-LV + 5%PHT + 1%liquid lubricant + barite. Three key treatment agents were selected as the investigation objects, namely inhibitors KCI, fluid loss reducers JT888, and coating agents IND10. The added amount of each treatment was used as the input, and a support vector machine model with *AV*, *PV*, *FL*_API_, and *R* as the output was established, respectively. Its structure is shown in Figure 5.

Through experiments, *AV*, *PV*, *FL*_API_, and *R* of drilling fluids of 50 groups of the above 3 treatment agents were measured in different dosages and combinations. Forty groups of data were randomly selected as SVM model training samples, and the remaining 10 groups of data were used as model test samples. Experimental data are listed in Table 2.

Use the remaining 10 groups of experimental data to check the predictive ability of the model, and the mean squared error (MSE) is commonly used in the support vector machine to measure the predictive accuracy of the training gained model, and the MSE calculation formula is (19). The smaller the value of MSE, the better the accuracy of the prediction model in describing the experimental data.  Table 3 compares the prediction results of the model with the experimental results.

(19) $$\begin{array}{c} MSE=\frac{1}{n}\overset{n}{\underset{i=1}{\sum }}\;{\left(x_{m}-x_{p}\right)}^{2}\text{.} \end{array}$$

In the formula

 
*x*_*m*_-experimental test value;
 
*x*_*p*_-parameter values for predicting performance.

From Table 3, it can be seen that the model established by the support vector machine to predict the performance parameters of the drilling fluid has high prediction accuracy and can meet the requirements of drilling fluid design. It can be used to build the subsequent drilling fluid formulation optimization design model.

**Figure 6:**
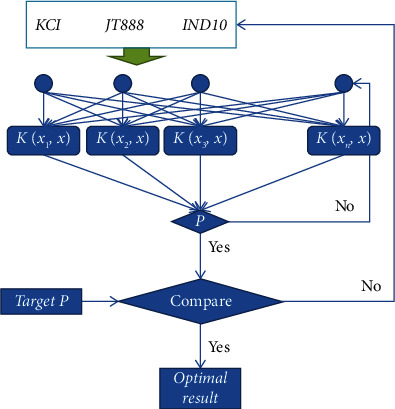
Drilling fluid formulation optimization design model structure.

On the basis of obtaining the SVM prediction model of drilling fluid performance parameters, this prediction model is used as a part of the model for inversion of the treatment agent dosage in the entire drilling fluid formula, and the drilling fluid performance required in different situations is used as the target parameter. The dosages of KCI, JT888, and IND10 are calculated by inputting the control variables into the prediction model. If the error between the output results of the prediction model and the target parameters is within the allowable range, it is considered that the dosages of the three treatment agents at this time can meet the performance requirements of the drilling fluid and output the result of adding this group. The computational structure model is shown in Figure 6.

A calculation example is as follows.

### 4.1. Drilling Fluid Formulation Design

Under the drilling fluid formulation optimization design model, the *AV*, *PV*, *FL*_API_, and *R* of the drilling fluid (40 mPa^*∗*^*s*, 37.0 mPa^*∗*^*s*, 4.2 mL, and 85.0%, respectively) are treated as the target performance parameters of this drilling fluid. The commonly used dosages of KCl, JT888, and IND10 are 0–20.0%, 0–2.0%, and 0–2.0%, respectively, which are the trial calculation ranges, and this model is used for calculation. If the errors of the calculated *AV*, *PV*, *FL*_API_*R,* and the target performance parameters are within 5%, 5%, 3%, and 5%, respectively, the requirements of the target performance parameters are met. At the same time, the amount of treatment agent reversed by the model is output.

Under the given calculation step, the model calculates a total of 9238 sets of data. Excluding some formulas with excessive addition, the formulas that meet the error range are shown in Table 4.

### 4.2. Experimental Verification Model

Although the support vector machine has good generalization ability, it can be seen from the error data of the previous prediction model establishment and test that its prediction accuracy also has a certain deviation, so the experimental verification is carried out on the reversely deduced treatment agent dosage formula. The experimental results are shown in Table 5.

It can be seen from the above chart that under the SVM model, a target drilling fluid performance may obtain a variety of drilling fluid formulations that meet the requirements, of which groups 1 and 3 are the preferred formulations, and their SVM calculation results are similar to the experimental results. However, there may also be unqualified treatment agent dosages. As can be seen in Figure 6, the AV and PV of groups 5 of treatment agents have a large gap with the target parameters after experimental verification, and they are unsatisfactory formulas.

## 5. Conclusions

In order to improve the quality of drilling fluid design, using computer to assist the design and introducing artificial intelligence system into the design is a common method to solve these shortcomings in the traditional drilling fluid design. At the same time, with the rapid development of oil and gas exploration and development technology and the increasing demand, modern drilling technology has put forward newer and higher requirements for drilling fluid, and various new drilling fluid technologies have been applied and developed. Today, in pursuit of high efficiency and low cost, intelligent drilling fluid design and management technology has also received more attention. Therefore, it is necessary to develop more practical software for modern drilling fluid design and drilling fluid data management. This paper introduces the basic theory of support vector machine and the principle of regression classification in detail, and analyzes and explains the two difficult problems of support vector machine kernel function selection and kernel parameter determination. Finally, the method of SVM applied to drilling fluid formulation design is studied, and a SVM model for predicting drilling fluid formulation is constructed, and it is verified by experiments that the model has good prediction accuracy.

## Figures and Tables

**Table 1:** Model prediction accuracy.

Model	LK	PK	RBF	SK
Accuracy (%)	74.88	cannot fit	97.31	85.36

**Table 2:** Experimental data.

Number	Dosage of key treatment agent (%)			The performance parameters of drilling fluid obtained from the experiment			
	KCl	JT888	IND10	AV (mPa^*∗*^*s*)	PV (mPa^*∗*^*s*)	FLAPI (mL)	*R* (%)
1	0	0	0	29.12	27.12	7.02	37.92
2	0	0	0.5	37.29	29.79	6.49	54.79
3	0	0	1.0	45.13	38.13	6.63	67.13
4	0	0	1.5	50.79	45.79	6.19	78.79
5	0	0.5	0	33.12	26.12	5.22	38.12
6	0	0.5	0.5	37.79	30.79	4.69	54.79
7	0	0.5	1.0	47.13	40.13	5.13	70.13
8	0	0.5	1.5	48.79	41.79	4.79	80.79
9	0	1.0	0	39.12	31.12	4.72	39.12
10	0	1.0	0.5	39.79	31.79	4.19	55.79
11	0	1.0	1.0	46.13	39.13	4.53	68.13
12	0	1.0	1.5	52.79	48.79	3.99	81.79
13	0	1.5	0	46.12	37.12	3.32	41.12
14	0	1.5	0.5	46.79	38.79	2.99	55.79
15	0	1.5	1.0	49.13	40.13	3.23	69.13
16	0	1.5	1.5	51.79	45.79	2.79	82.79
17	3	0	0	29.12	27.12	7.02	46.12
18	3	0	0.5	37.79	28.79	6.59	56.79
19	3	0	1.0	45.13	38.13	6.93	68.13
20	3	0	1.5	49.79	45.79	6.49	83.79
21	3	0.5	0	33.12	25.12	5.22	44.12
22	3	0.5	0.5	37.79	30.79	4.89	57.79
23	3	0.5	1.0	46.63	41.13	5.13	71.13
24	3	0.5	1.5	47.79	40.79	4.79	85.79
25	3	1.0	0	38.12	31.12	4.62	43.12
26	3	1.0	0.5	38.79	30.79	4.19	58.79
27	3	1.0	1.0	45.13	38.13	4.63	72.13
28	3	1.0	1.5	51.79	46.79	4.19	82.79
29	3	1.5	0	44.12	36.12	3.32	46.12
30	3	1.5	0.5	46.79	37.79	2.99	54.79
31	3	1.5	1.0	49.13	39.13	3.23	74.13
32	3	1.5	1.5	51.79	45.79	2.89	80.79
33	5	0	0	29.12	26.12	7.02	50.12
34	5	0	0.5	36.79	28.79	6.69	58.79
35	5	0	1.0	43.13	35.13	6.93	73.13
36	5	0	1.5	47.79	42.79	6.59	84.79
37	5	0.5	0	34.12	25.12	5.12	58.12
38	5	0.5	0.5	37.79	31.79	4.79	59.79
39	5	0.5	1.0	45.13	38.13	5.23	73.13
40	5	0.5	1.5	46.79	38.79	5.09	87.79
41	5	1.0	0	37.12	30.12	4.52	50.12
42	5	1.0	0.5	37.79	28.79	4.39	58.79
43	5	1.0	1.0	44.13	36.13	4.33	75.13
44	5	1.0	1.5	49.79	43.79	4.09	84.79
45	5	1.5	0	44.62	34.12	3.32	50.12
46	5	1.5	0.5	44.79	36.79	3.29	58.79
47	5	1.5	1.0	47.13	37.13	3.23	73.13
48	5	1.5	1.5	49.79	42.79	3.09	81.79
49	7	0	0	27.12	25.12	6.92	51.12
50	7	0	0.5	35.79	27.79	6.69	60.79

**Table 3:** Model prediction error table.

Error	AV	PV	FL_API_	*R*
Mean squared error	1.23	1.65	1.32	0.26
Maximum error (%)	6.02	6.71	4.24	2.82

**Table 4:** Calculation result of drilling fluid formulation optimization design model.

Number	Recipe calculated from the optimized design model			Corresponding drilling fluid performance parameters			
	KCl (%)	JT888 (%)	IND10 (%)	AV (mPa^*∗*^*s*)	PV (mPa^*∗*^*s*)	FL_API_ (mL)	*R* (%)
1	2.25	0.59	0.68	41.41	36.95	4.19	88.18
2	3.68	0.44	1.93	39.82	36.78	4.64	91.93
3	5.21	1.09	0.88	42.13	38.71	3.59	89.48
4	9.25	1.24	0.73	41.25	37.15	4.04	89.23
5	6.68	0.69	0.18	40.48	36.48	4.09	88.48

**Table 5:** Experimental results.

Number	Recipe calculated from the optimized design model			Corresponding drilling fluid performance parameters			
	KCl (%)	JT888 (%)	IND10 (%)	AV (mPa^*∗*^s)	PV (mPa^*∗*^s)	FL_API_ (mL)	*R* (%)
1	2.25	0.59	0.68	41.72	38.25	4.29	89.88
2	3.68	0.44	1.93	50.01	44.98	5.44	90.93
3	5.21	1.09	0.88	43.84	40.51	3.09	91.88
4	9.25	1.24	0.73	40.52	35.75	4.04	88.33
5	6.68	0.69	0.18	38.77	35.78	4.19	88.58

## Data Availability

The dataset can be obtained from the corresponsing author upon request.
